# Generating and Detecting Solvable Chaos at Radio Frequencies with Consideration to Multi-User Ranging

**DOI:** 10.3390/s20030774

**Published:** 2020-01-31

**Authors:** Aubrey N. Beal, Seth D. Cohen, Tamseel M. Syed

**Affiliations:** 1 Department of Electrical and Computer Engineering, University of Alabama in Huntsville, Huntsville, AL 35899, USA; ts0109@uah.edu; 2 Southern Research, Birmingham, AL 35255, USA; scohen@southernresearch.org

**Keywords:** chaos, spread spectrum, matched filter, collision avoidance, mutual interference, crosstalk, FMCW, chaotic radar, noise radar

## Abstract

High entropy waveforms exhibit desirable correlation properties in radar and sonar applications when multiple systems are used in close proximity. Unfortunately, the information content of these signals can impose high sampling requirements for digital detection techniques. Solvable chaotic oscillators have been proposed to address such issues due to their simple, matched filters, where hardware has been demonstrated with a bandwidth of 10–20 kHz. To extend applications of these systems, we present theory, design, and experimental verification of solvable chaos at 1 MHz using simple off-the-shelf components. The waveforms produced by this system were propagated over a 2.45 GHz RF link and detected with an RLC-based, purely analog matched filter. Further, we show that properties of this special class of chaotic systems can be exploited to yield RF noise sources that are generally advantageous for multi-user ranging applications when compared to conventional techniques. The result is a simple, low-cost, and potentially low-power RF ranging system that requires very little digital signal processing.

## 1. Introduction

Crowded sensors must coexist in a local environment and share resources. This crowding compounds as interconnected devices become ubiquitous [1] and concepts like the Internet of Things (IoT) mature [2]. Ranging sensors that support multiple users in close proximity are key to enabling emerging technologies like wireless sensing in healthcare [3], autonomous transportation [4], and drones as a service [5]. Unfortunately, signal detection issues manifest when many ranging sensors share a local environment.

As an example, automotive collision avoidance radars commonly use frequency modulated continuous wave (FMCW) schemes that can suffer from mutual interference issues [6,7,8]. As interference increases with multiple users, the reliability of assisted driving systems can degrade [9]. These issues motivate the use of specialized, less-predictable waveforms [10,11,12,13,14,15]. Thus, we propose the use of solvable chaos as it exhibits noise-like properties and may be optimally detected using a simple matched filter [16].

**Figure 1:**
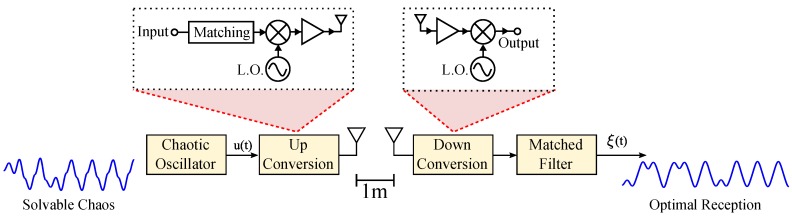
Radio frequency experiment overview showing the wireless propagation and optimal detection of solvable chaos.

Solvable chaos also has had limitations, and overcoming some of those barriers will be the main focus of this article. The hybrid nature of solvable chaos paired with the aim for higher operational frequencies makes electronic implementation difficult [17]. As a result, physical implementations have historically contained deviations from the ideal analytic solution at frequencies above ranges of 200 kHz [18]. In this manuscript, we will examine a circuit representation of a technique for mitigating these design issues. As an example of our efforts, we showcase a solvable chaotic oscillator using consumer-off-the-shelf (COTS) components at 1.11 MHz. This example is then experimentally up-converted at 2.45 GHz, sent through a wireless channel over a distance of 1 meter, and received using a simple RLC matched filter. A pictorial representation of this experiment is shown in Figure 1.

We intend for the results presented here to serve the community in two ways. First, we hope that the validation an RF circuit implementation of solvable chaos, which has been properly compensated and detected over a wireless channel, will help the community to consider applications for solvable chaos as wireless sensors. Second, we show that solvable chaos is generally suitable for multi-user range sensing. This has been previously shown in hardware at ≈10 kHz by via an acoustic ranging demonstration that successfully ranged a direct path link while an identically fabricated chaotic oscillator corrupted the channel [19]. Through simulation, we show that for the scenario of more than one chaotic interferer, behavior is in accordance to the findings in [19], even in the worst-case scenario of all users being phase-locked.

Last, we note that the concept of this ranging scheme and the mathematics that prove the benefits of solvable chaos have been published in the literature for close to a decade. This raises the question, why has solvable chaos not yet been transitioned more widely into practice? Overall, we believe there are two answers to this question: (1) chaotic waveforms are exotic/unconventional and require a thorough understanding of both nonlinear dynamics and electronics to fabricate, and (2) even with this understanding, technical hurdles degrade the performance of solvable chaotic oscillators at higher frequencies. Thus, along with the goal of demonstrating high-frequency solvable chaos, we aim to provide the initial foundation for readers to move beyond answering question (1) to focus their interest/efforts on answering question (2).

## 2. Background

Ranging systems that support multiple users in close proximity are key to enabling emerging technologies as the IoT application space matures. Unfortunately, unpredictable environments are a major obstacle for ranging applications in transportation, space exploration, logistics, and disaster relief and rescue [20]. Several layered approaches and sensing modalities are often needed to ensure correct operation over a wide range of conditions. For instance, automotive collision avoidance systems often employ combinations of radar, sonar, lidar, and cameras, especially in degraded visual environments [7,21,22]. The complexity of these types of approaches, however, often requires stringent design specifications, and maintaining those specifications can often be difficult in multi-user settings.

In this section, we provide background for some complications that occur when many systems make distance measurements in a crowded environment. Particularly, multiple FMCW ranging systems employed by different users in a shared environment can generate cross-talk or mutual interference that degrades sensing abilities. This issue is common in many realistic scenarios involving vehicles [6,7,8,9], mobile robots [23,24], and drones [25,26,27]. One successful mitigation technique requires user-specific [15], entropic (noisy) waveforms for spread spectrum techniques such as those used in sonar [10,11] and radar [12,13,14,28] systems. Throughout this section, we provide a brief description of the limitations of FMCW ranging in order to motivate the use of user-specific, noisy waveforms generated from a solvable chaotic source. As we will demonstrate later, a primary advantage to using solvable chaos is the use of simple RLC circuits to optimally detect noise-like waveforms in the presence of noise and other interfering sources.

### 2.1. FMCW Limitations

Radar and sonar systems detect the presence of objects by observing returning echoes from the environment [29,30]. These echos contain transmitted information such as pulses or continuous-waves (CW). The Doppler Effect is the basis for continuous-wave (CW) ranging. Generally, CW schemes are advantageous when compared to other methods (such as pulsed systems) due to their simplicity, peak-power requirements [29] and low cost.

**Figure 2:**
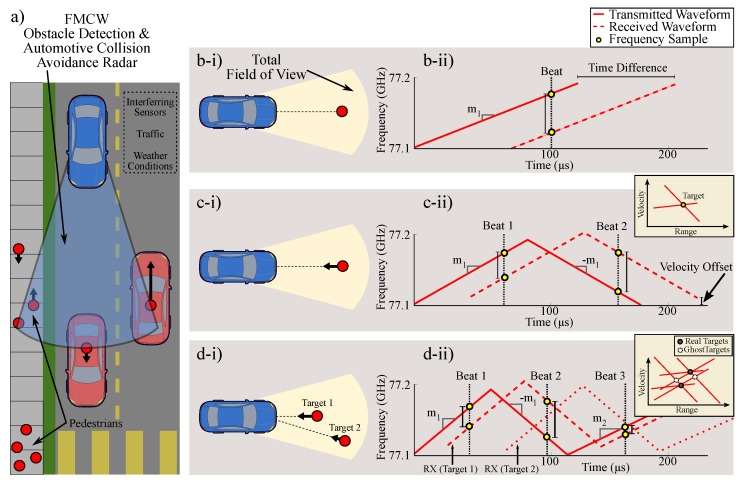
(**a**) Illustration of a congested, sensing environment with multiple users and mutual interference due to FMCW radar range measurements. (**b-i**) Detection scenario for FMCW scheme with a linear chirp that can detect a single, static target by measuring a beat frequency. (**b-ii**) Ideal, radar transmit (red, solid) and receive (red, dotted) waveforms for a single up-ramp chirp transmission for ranging a single static target. (**c-i**) Detection scenario with a single, moving target at constant velocity. (**c-ii**) Ideal, radar transmit (red, solid) and receive (red, dotted) waveforms for an up-ramp and down-ramp chirp transmission for ranging a single target moving at a constant velocity. (**d-i**) Detection scenario with multiple, moving targets. (**d-ii**) Ideal, radar transmit (red, solid) and receive (red, dotted) two waveforms with two up-ramps and a down-ramp chirp transmission for ranging two moving targets.

An FMCW system is a CW system with increased bandwidth in the transmitted signal. This modification allows for more information to be collected about targets in the environment. Typically, as the complexity of waveforms used to modulate ranging signals increases, the amount of information detected about targets in that environment also increases. This is illustrated by FMCW sensing of multiple targets as depicted by Figure 2.

These ranging systems are popular due to their simplicity and low cost; however, issues can emerge. An important example is found in the case of automotive collision avoidance systems as illustrated in Figure 2a [31]. Automotive FMCW radars are favored for collision avoidance due to their performance in poor weather conditions [32] and ability to accurately measure ranging when compared to passive sensors [33]. However, clutter and unwanted signals, like those from other FMCW radars, challenge such applications due to the erroneous appearance of *ghost* targets as depicted in the range-velocity plots given by Figure 2.

Multiple user interference issues, like ghost targets, develop because radar enabled vehicles in heavy traffic tend to jam one another when FMCW schemes are used [6]. Ultimately, this problem occurs because many systems must share a limited natural resource: the electromagnetic spectrum. Thus, the need for user-specific waveforms is motivated by the combined effects from both mutual interference issues due to multiple sensors and multitarget returns problems [15].

Skolnik discusses FMCW waveform restrictions for multiple targets in detail [29]. We note that the effects for mutual interference are similar. Interestingly, as the number of interferers and/or targets in a crowded/local sensing environment increases, a need for user-specific, noise-like waveforms becomes clear. This need is illustrated as noted in [29] by Figure 2.

As a progression from the simple case of a single, stationary target is made to multiple, moving targets, the modulation used for FMCW chirps must increase in complexity to correctly discard ghost targets via postprocessing. Generally, the FMCW output will contain more frequency content as more targets are present. For linear systems, each target may be determined by measuring individual beat frequency components [29], given that they may be separated by signal processing techniques such as filtering or Fast Fourier Transforms (FFTs).

Eventually, widespread use of simple FMCW ranging techniques for radar and sonar begins to resemble an electronic battlefield when many local users are introduced [6]. Denial and deceptive jamming are both present in heavy traffic scenarios and contribute to poor signal-to-noise ratio (SNR) and the generation of ghost targets. Mitigating these issues is vital for many autonomous vehicle tasks such as pedestrian detection [34].

There is a clear need for the use of user-specific waveforms and receivers that maximize SNR result. Some solutions involve complex waveforms, jamming avoidance, and spread spectrum properties [6]. Additionally, researchers have shown that waveforms can be created using random signals, encryption techniques, or spectrum sharing algorithms [35] that reduce the probability of mutual interference.

As documented through the literature, noisy (random) waveforms reduce mutual interference, but they are often difficult to engineer for applications in sensor technologies. For example, pseudorandom codes demand on-board signal processing resources and draw additional power due to Nyquist sampling limits. These challenges are of particular concern in receiver designs, which must conventionally sample twice the full bandwidth of the noisy waveforms to obey the Nyquist criteria. Further, in typical noise radars with range resolutions below 1m, sampling rates can reach the GHz range. Last, we acknowledge new and interesting ranging topologies that utilize partially coherent sources to allow for increased accuracy when additional bandwidth is not available [36]; similar to noise-based ranging, a random or pseudorandom source is the backbone of these ranging waveforms.

Of interest to this manuscript, chaos is a random-like waveform that can be reliably produced in electronic systems [37]. By using chaos, ranging systems effectively use signals with high information content similar to stochastic noise. The frequency content of these *noisy* signals is spread across a wide range. This reduces the potential for interference because transmission energy is located at many frequencies that correspond uniquely to a specific transmitted signal.

Overall, the sampling and noise production constraints seemingly require more power, memory and sensitivity compared to conventional techniques such as FMCW. As we will demonstrate, chaos is a mathematical concept for processes that can generate noise-like waveforms, and certain chaos has beneficial properties over even conventional systems when exploited in applications of ranging systems.

### 2.2. Solvable Chaos

Weather modeling famously led to the discovery of chaotic dynamics that give unpredictable behavior despite their deterministic properties [38,39]. Later, discoveries showing the synchronization [37] and control [40] of chaotic systems motivated chaos-based communication schemes. The behavior of the Lorenz system was eventually viewed through the framework of communications theory [41]. It was shown that waveforms from the Lorenz system may be used to construct a basis function similar to those used in binary phase-shift keying (BPSK) waveforms. However, the basis functions for the Lorenz system are non-causal, grow exponentially, and are not fixed [42]. Only within the last decade, researchers have successfully engineered simplified versions of these basis functions. As a result, a new class of systems that produce solvable chaos demonstrates important and surprising results [16,43]; they allow for elegant detection schemes in ranging systems using only inexpensive, low-power hardware.

In order to provide context for these systems, two core requirements of an ideal receiver design have been assumed [43]. First, suppose we wish to design a receiver that is simple, such that cost and power are reduced. To do so, we limit our designs to use only three basic components: a resistor, an inductor, and a capacitor (RLC). Importantly, this satisfies a reasonable lumped element approximation for all passive transmission lines. Second, suppose we wish to construct an optimal receiver such that the SNR of the received waveform is maximized; this requirement is known as a matched filter.

The notion of such extremely simple, optimal receiver designs was once thought to be impossible due to their infinite impulse response and inter-symbol interference properties [44]. The concept was historically dismissed as *not realizable* [44,45]. Presently, matched filtering is conventionality performed as a postprocessing technique by digital signal processors (DSPs) [30]. This is because matched filtering requires a convolution with the time-reversed version of the waveform, a process that it is easy to implement in software. A DSP approach engineers a digital filter for the a given waveform. However, in our case, we have already assumed a general RLC filter, and with this assumption, we instead engineer a source to produce specialized waveforms such that the RLC filter provides the time-reversed convolution needed for a matched filter.

New and exciting results show that waveforms matched to general RLC filters must *necessarily* be chaotic [43]. This remarkable outcome draws a deep connection from the field of nonlinear dynamics to modern communication and signal detection theory. Further, it has been shown that these particular nonlinear systems exhibit an unusual property: they have closed-form analytic solutions. Examples in hardware have been demonstrated experimentally through circuit realizations in the kHz frequency range [16].

As we will show in the next section, the closed-form solution of a solvable chaotic system manifests as a conventional communication waveform with several beneficial properties. First, it permits a compressed sampling (sub-Nyquist) scheme that requires less memory and power when compared to other noise sources. Second, the closed-form solution allows for the use of common engineering tools to predict metrics such as: (i) expected frequency content, (ii) probability density [46], (iii) entropy [16], (iv) ambiguity surface [47], and (iv) bit error rate [16] for the system. These benefits are unique to this special class of solvable chaotic systems because analytic descriptions of such quantities in general can only be assessed empirically for other chaotic systems.

#### 2.2.1. Solvable Chaos Theory

Solvable chaos may be constructed by considering the following unstable, system of equations.

(1) $$\begin{array}{c} \begin{array}{cc} {\dot{u}}_{1} & =u_{2}\\ {\dot{u}}_{2} & =2\beta u_{2}-\left(\omega^{2}+\beta^{2}\right)\left(u_{1}-s\right), \end{array} \end{array}$$

where 0<β≤ln(2) and ω=2π with initial conditions $u_{1}\left(0\right)$, $u_{2}\left(0\right)$, and s(0). This system tends to grow exponentially without bound. To limit this growth, a forcing function *s* is applied dynamically such that s(t) has transitions set by a *guard condition*

(2) $$u_{2}=0\Rightarrow s\left(t\right)=\mathrm{sgn}\left(u_{1}\right),$$

where the signum function sgn(u) is defined as

(3) $$\mathrm{sgn}\left(u\right)=\left\{\begin{array}{cc} +1, & u\geq 0\\ -1, & u<0. \end{array}\right.$$

**Figure 3:**
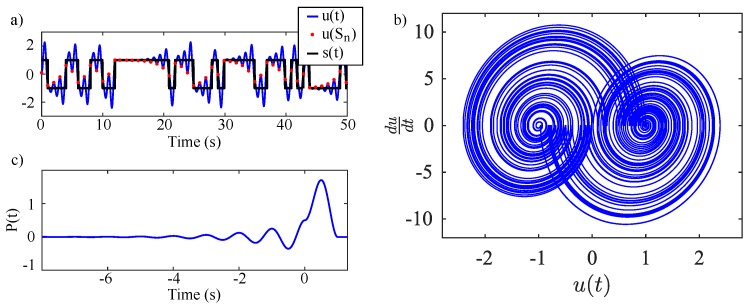
(**a**) MATLAB simulation of typical waveforms generated by the solvable chaotic oscillator with β=ln(1.99) with initial conditions $u_{1}\left(0\right)=0.1$, $u_{2}\left(0\right)=0$ and s(0)=1. (**b**) Typical phase portrait of a solvable chaotic oscillator with β=ln(1.99). (**c**) Basis pulse for solvable chaotic oscillator with β=ln(1.99).

A typical solution consists of an unstable harmonic oscillation that grows exponentially about a bias or set point. This growth continues until $u_{1}$ crosses zero. Upon the next zero crossing of $u_{2}$, the set point is forced to take one of two values given by $\mathrm{sgn}(u_{1})$, where the oscillation resets and then grows about the new set point. This results in globally bounded behavior despite negative damping. A typical waveform and phase portrait given by the oscillator is shown in Figure 3a,b.

**Figure 4:**
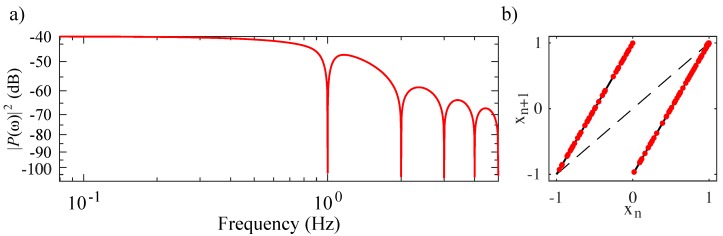
(**a**) Power spectral density of the chaotic waveform (red solid). (**b**) Iterated map illustrating the Bernoulli shift map behavior of the chaotic oscillator with $u\left(t_{sn}\right)=x_{n}$ as red scatter points where $t_{sn}$ corresponds to a symbol $S_{n}$ occurring at a guard condition trigger event. The equation for this iterated map is given by Equation (10).

A single, fixed basis function P(t) for the system’s solution may be found by solving for the unit pulse response of Equation (1) for a given set point. This gives a function with boundary conditions $P\left(T\right)=0,P\left(-\infty \right)=0,P\left(0^{-}\right)=P\left(0^{+}\right)$ resulting in

(4) $$P\left(t\right)=\left\{\begin{array}{cc} \left(1-e^{-\beta T}\right)e^{\beta t}\left[\cos\left(\omega t\right)-\frac{\beta }{\omega }\sin\left(\omega t\right)\right] & t\leq 0\\ 1-e^{\beta (t-T)}\left[\cos\left(\omega t\right)-\frac{\beta }{\omega }\sin\left(\omega t\right)\right] & 0\leq t\leq T\\ 0 & t\geq T, \end{array}\right.$$

where the period T=1 and frequency ω=2π can be assumed for simplification. The resulting basis function is shown by Figure 3c. The basis pulse is non-causal with an infinitely long precursor tail. This precursor signifies the determinism of the system. The envelope of the power spectral density of the oscillator’s output signal is the square of the Fourier transform of the system’s basis function [42]

(5) $$P\left(\omega \right)=\frac{1-e^{-j\omega T}}{j\omega }\cdot \frac{\beta^{2}+\omega_{o}^{2}}{{\left(\beta -j\omega \right)}^{2}+\omega_{o}^{2}},$$

and is shown in Figure 4.

This expression serves as an example of a useful engineering specification that may be obtained by exploiting the chaotic waveform’s solvability. The analytic spectrum is unique to this solvable system and may be used to test and evaluate engineering metrics. In practice, these metrics can describe the fidelity of a circuit implementation, inform channel requirements or assist the design of antennas.

The complete solution for $u_{1}$ may be expressed analytically in terms of P(t) by combining many basis pulses that add constructively and destructively resulting in a hybrid, chaotic waveform. The resulting solution takes the form

(6) $$u_{1}\left(t\right)=\sum_{m=-\infty }^{\infty }s_{m}\cdot P\left(t-m\right),$$

where $s_{m}$ are symbols that represent an unpredictable bit stream related to the system’s initial conditions. Interestingly, this resulting waveform is constructed from inter-symbol interference and takes a familiar form used to express communication signals like BPSK [16]. Again, this expression allows for physical implementations to be evaluated by their deviation from what is analytically expected.

Further, Corron et al. [16] showed that the system output contains embedded dynamics of the Bernoulli shift map. This mapping allows simple analysis of the available entropy and under some cases this map allows for analysis using symbolic dynamics [48,49,50]. Consider the switching events n≤t<n+1 that arise from triggering the guard condition. A shift map description may be obtained by considering the general solution $u_{1}$ between these events

(7) $$u_{1}\left(t\right)=s_{n}+\left(u_{1}\left(n\right)-s_{n}\right)e^{\beta (t-n)}\left(\cos\left(\omega t\right)-\frac{\beta }{\omega }\sin\left(\omega t\right)\right).$$

The guard condition is met when $u_{2}$ is zero

(8) $$u_{2}\left(t\right)=-\frac{\omega^{2}+\beta^{2}}{\omega }\left(u_{1}\left(0\right)-s_{0}\right)e^{\beta t}\sin\left(\omega t\right)=0,$$

with $u_{1}\left(0\right)$ and $s_{0}$ as initial conditions.

Evaluating Equation (8) shows the progression of a map by instances in which the guard condition is met (t=1/2,1,...) [16]. This relationship gives

(9) $$u_{1}\left(n+1\right)=e^{\beta }u_{1}\left(n\right)-\left(e^{\beta }-1\right)s_{n}.$$

For β=2, the resulting map is commonly written as

(10) $$x_{n+1}=2x_{n}\mathrm{mod}\left(1\right),$$

and is referred to as a *shift map* shown in Figure 4b. This map can be defined to act on the interval $-1\leq u_{n}\leq 1$ or $0\leq u_{n}\leq 1$. Points outside these intervals are either fixed or globally unstable. This relation gives discrete, chaotic behavior that can be described in terms of the current iterate value $u_{n}$ and bit representation of the initial condition $s_{n}$ by

(11) $$x_{n+1}=2x_{n}-s_{n}.$$

The one-dimensional map representation of this system not only gives criteria (such as entropy and simple analysis of allowed dynamics) to engineer towards, but also provides a different perspective of these systems as entropy sources. This is particularly important for highlighting that multiple (even identical) copies of these systems will naturally produce statistically orthogonal bit streams suitable for noise radar and random number generation. In other words, pseudorandom codes and sources are not required. Furthermore, it is important to acknowledge that despite the deterministic properties of this system, it acts as an information source by amplifying small uncertainties for a given initial condition. Even in a noise-free environment, this system would generate information due to limited precision and available memory for describing the initial condition. This quality is at the heart of analog chaotic systems and is not found in FPGAs (or other computational approaches) that produce simulated chaotic waveforms based on the output of finite state machines.

For the shift map, this property is illuminated by considering initial conditions selected from a continuum on the unit interval expressed as

(12) $$x_{0}=\frac{1}{2}\sum_{i=0}^{\infty }s_{i}2^{-i}=0.s_{0}s_{1}s_{2}...$$

where each bit is represented by a symbol $s_{i}\in \left\{0,1\right\}$. This expression represents a binary form of a given initial condition $u_{1}\left(0\right)$. The action of the shift map described by Equation (10) is clear when considering this binary representation because each iteration multiplies $x_{n}$ by 2 which shifts the symbols to the left. The leading symbol $s_{n}$ is shifted to the left of the decimal to give $x_{n+1}^{\prime }=s_{n}.s_{n+1}s_{n+2}s_{n+3}...$ The mod(1) operation limits $x_{n+1}\leq 1$ by discarding $s_{n}$ such that $x_{n+1}=0.s_{n+1}s_{n+2}s_{n+3}...$ This process limits each iterated value to the unit interval.

In practice, initial conditions provided by some noise source or heat bath continuum are guaranteed to be irrational and can only be measured to some finite precision [51]. The positive slope of this map will amplify the uncertainty of this measurement at each iteration [52]. Although this uncertainty yields unpredictable behavior after a few iterations, the map’s functionality is deterministic and an exact analytic solution for the n^th^ state of the system may be described by Equation (10) acting on the symbol sequence in Equation (12).

Essentially, the map operates on an initial condition by continually amplifying the state of the system and using a modular function to keep the state bounded. After each iteration, a new and eventually unknown value at the measurement limit is introduced. This provides a small deviation that is increased as the iteration continues, thus giving the system sensitivity to small changes. This action gives essentially random behavior with deterministic dynamics, a common property to all chaotic systems. A Lyaponov exponent, λ, which describes the exponential rate of this sensitivity, may be derived from this action resulting in a λ value equivalent to the slope (or gain) of the map.

The result governed by Equation (9) produces a constant slope of $e^{\beta }>1$. This positive slope/gain implies that the system is entropic and chaotic [16]. Thus, the system described by Equations (1)–(3) has a Lyapunov exponent λ=β, and the discrete values of $s_{n}$ correspond to symbols derived from the map shown in Figure 4. These symbols may be expressed as the iterates $u_{1}\left(n\right)$ (shown in red scatter points) relative to a partition. Furthermore, there exists a closed expression for the symbols [48] of this system [16] that allows for information encoding [42] and small perturbation control [40] through analytic methods for the realization of a chaotic radar/communication schemes with specific, optimal sequences, such as Barker codes, that give minimum correlation side-lobes in correlation receivers [53].

In the next section, we provide the theory and context for our assumed simple, yet optimal receiver. This treatment illustrates the practical advantage of a chaotic system with a closed-form solution that involves a fixed, basis function.

#### 2.2.2. Simple, Optimal Detection

Here, we outline specifically how solvable chaotic systems allow for optimization in regards to signal detection characteristics when considering SNR. Generally, improved SNR enhances the ability to perform object detection, proximity measurement, and tracking, with examples that include synthetic aperture imaging [30], remote sensing, and diagnostic sonography [54]. Assuming a fixed probability of a false alarm, the probability of a correct detection increases monotonically with SNR [30]. Thus, as SNR increases, desired performance levels may be achieved with less transmitted power. Practically, this benefits designs by reducing signal processing operations, power consumption, and transducer requirements [30].

Linear correlation provides an optimal detection of transmissions in the presence of noise. More specifically, when a linear channel corrupts a transmission via additive Gaussian white noise (AGWN), the optimal detection method is found to be a linear correlation known as a matched filter receiver [55]. For a priori basis functions, the matched filter maximizes the SNR of a transmitted waveform and results in a minimized bit error rate (BER).

Thus, matched filters provide the mathematical equivalent of a linear correlation. This well-known result powerfully implies that the presence of any physical waveform, g(t), may be optimally detected when considering corruption due to AGWN by simply selecting a filter, $h_{\mathrm{opt}}\left(t\right)$, with an impulse response that is proportional to a time-reversed, time-delayed (by period *T*) version of g(t) such that

(13) $$h_{\mathrm{opt}}\left(t\right)=g\left(T-t\right).$$

**Figure 5:**
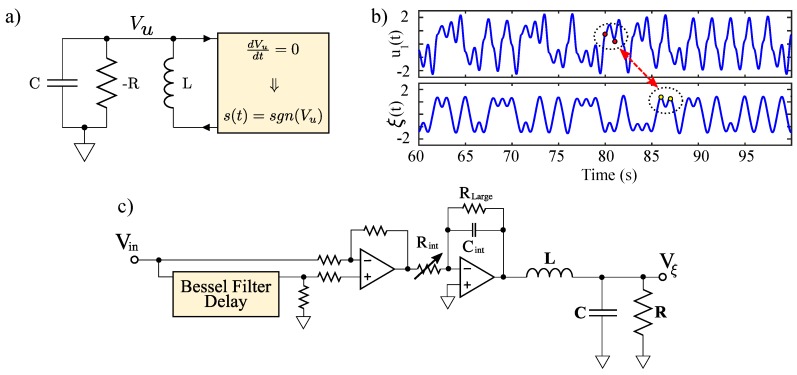
(**a**) High-level schematic of a solvable, chaotic oscillator circuit. (**b**) Matched filter operation with a SPICE simulated channel propagation delay of 5 unit time intervals with a transmitted signal u(t) from a solvable chaotic system given in (**a**) and the output of the matched filter in (**c**). (**c**) High-level schematic of a matched filter corresponding to the solvable, chaotic oscillator circuit given by (**a**).

The linear filter matched to the basis function P(t) in Equation (4) is then described by

(14) $$\dot{\eta }=v\left(t+1\right)-v\left(t\right),$$

(15) $$\begin{array}{c} \begin{array}{cc} {\dot{\xi }}_{1} & =\xi_{2}\\ {\dot{\xi }}_{2} & =\left(\omega^{2}+\beta^{2}\right)\left(\eta -\xi_{1}\right)-2\beta \xi_{2}, \end{array} \end{array}$$

where v(t) is the filter’s input, η(t) is an intermediate state, and ξ is the filter’s output. Equations (14) and (15) construct the matched filter for the basis pulse P(t) giving an impulse response of the optimal filter to be h(t)=P(T−t). Typical matched filter waveforms are shown later in Figure 5.

We note that the matched filter does not reconstruct the transmitted signal, but maximizes the SNR for the detection of the symbolic content contained within the transmitted signal. In the next section, we provide examples and introduce hardware designs that overcome practical issues when building these solvable chaotic generators in the MHz frequency ranges.

## 3. Hardware Design

Hardware realizations of solvable chaos with detection circuits have been realized at various frequencies up to ≈20 kHz [16,19,46,56,57]. Electronic generation of solvable, chaotic waveforms generally consists of mixed signal electronics that include an unstable, analog circuit and a digital switching circuit to implement the hybrid nature of system equations. Detectors for these waveforms utilize stable, matched circuits with integrator and delay stages to implement a correlation-based detection scheme. These arrangements have been achieved with simple, low-cost, and low-power hardware [16].

**Figure 6:**
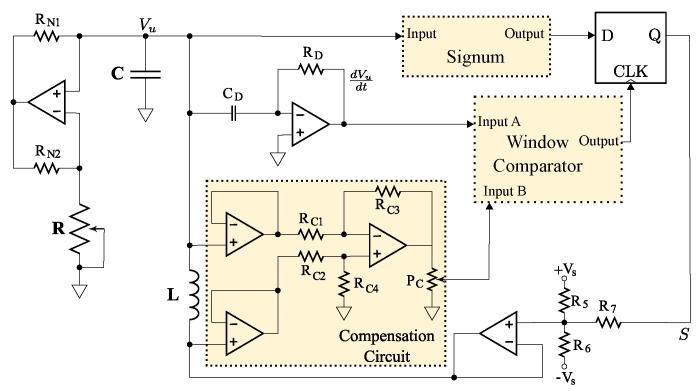
Schematic showing a circuit for generating high frequency, solvable chaos using COTS components. This arrangements includes compensation of finite switching times that result in delays around the circuit’s feedback loop.

Several design issues arise when generating high frequency solvable chaos. Circuit parameters of solvable chaotic oscillators must be preserved to ensure benefits from all of the analytical descriptions from the previous section and, particularly, for the matched filter receiver to function properly. This requirement imposes challenges as the frequency of these solvable chaotic systems is increased. A notably pertinent issue arises from imperfect digital switching in the circuits described in Figure 5 and Figure 6. Finite rise and fall times of digital switches are unavoidable and introduce unwanted propagation delays when signal content rivals the switching speeds. These issues combined with finite gain, bandwidth and slew rate in practical op-amps effectively limit the operational frequencies that may be achieved using these systems. Recently, switching compensation has been developed to mitigate some of these effects [17]. This compensation corrects imperfections in the circuit’s finite switching times, though issues can still arise from the analog amplification mechanisms [58,59].

### 3.1. Generating Solvable Chaos at Radio Frequencies

Chaotic oscillators generally require a stretching mechanism to achieve local instability and a folding mechanism to keep the system globally stable. To implement a hardware realization of the aforementioned solvable chaotic system, a circuit was designed to provide both the stretching and folding functionality found in chaotic systems. A schematic for implementing an electronic solvable chaotic oscillator is given by Figure 6.

The stretching is provided by a −RLC (negative resistance RLC) network that is used to realize a physical representation of the second order differential equation given by Equation (1). Practically, this was implemented using an op-amp negative impedance converter (NIC) with a discrete element inductor and capacitor. This provides a voltage $V_{u}\left(t\right)$ that corresponds to the theoretical solution given by $u_{1}\left(t\right)$.

Practical implementation of the folding mechanism involves a comparator that is triggered by zero crossings of $dV_{u}/dt$, which enables sampling of $V_{u}\left(t\right)$ after a signum function is applied as illustrated in Figure 6. This sampling is held in a D-latch with an input of a comparator (referenced to ground) which gives the sign of $V_{u}\left(t\right)$. This mechanism is a physical representation of the guard condition given by Equation (2).

Combined together in the circuit, the negative damping causes exponential growth of the output voltage that would normally reach the power supply rails if it were not monitored. The folding mechanism keeps these oscillations stable about the two fixed points and contributes to the system’s mixing properties. Details and issues relating the frequency increase of op-amp based NICs used for negative resistances in the −RLC have been addressed [58,59,60].

As noted earlier, the benefits of this system can degrade with unwanted delays between the stretching and folding mechanisms. This type of latency has been shown to cause distortion in the chaotic waveform, thus, compromising its solvability [17,18] in COTS implementations. As mentioned earlier, compensation methods have been theoretically examined to correct for these imperfections while retaining solvability [17]. In this manuscript, these compensation techniques are realized for the first time in hardware, where the compensation circuitry is shown in Figure 6. The circuit was fabricated as described in [16,17,18] with op-amps of type LT1361, 0805 passive components and *R* = 1.77 kΩ (variable through a 5 kΩ potentiometer), *L* = 48 mH and *C* = 330 pF.

**Figure 7:**
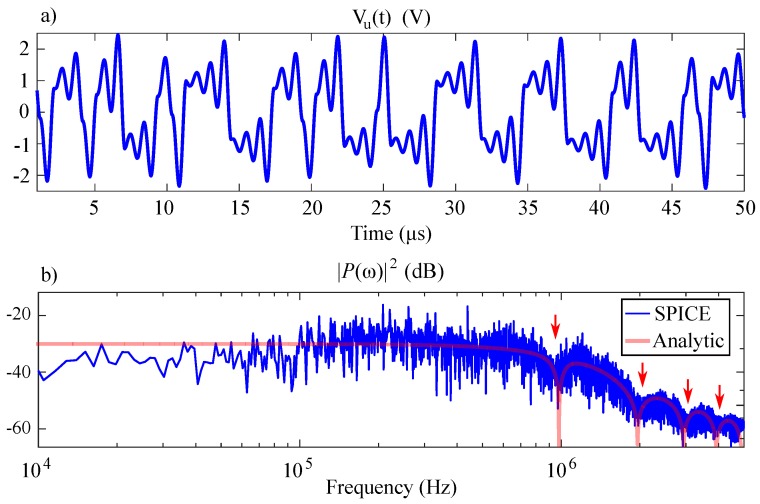
SPICE simulation of 1MHz chaotic oscillator: (**a**) time series and (**b**) spectrum.

The resulting time series and spectral simulation results for a ≈1 MHz simulation of this circuit using LTSPICE IV are shown by Figure 7. These results show that the spectral output from the circuit implementation given in Figure 6 has the same characteristic nulls as the analytic expression given by Equation (5).

### 3.2. Matched Filter Detection

A hardware implementation of the matched filter given by Equations (14) and (15) was constructed according to the schematic shown in Figure 5. In the figure, the input signal and its delayed copy are presented to a difference amplifier. This difference is integrated to give the intermediate state η(t). The intermediate state is then presented to a RLC network that performs the time-reversed convolution as the counterpart to the −RLC of the chaotic signal source.

A 2 kΩ potentiometer was used to adjust the integration constant of the intermediate stage with $C_{int}=10$ nF and $R_{Large}=6$ kΩ. The RLC tank circuit was set to match the −RLC tank at the chaotic transmitter. The values were 48 μH, 470 pF and a 1 kΩ variable resistor set to give 1.561 kΩ measured using a Fluke 179 true RMS multimeter.

**Figure 8:**
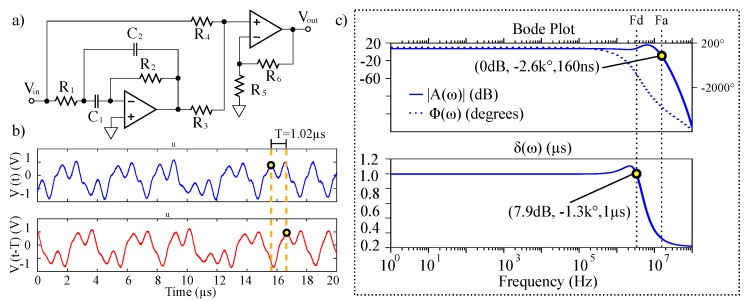
(**a**) Schematic of a single delay cell for the Bessel–Thompson filter circuit. (**b**) Hardware data showing (**a**) a chaotic input signal and (**b**) the output of the Bessel–Thompson filter delaying the chaotic input signal by 1.02 μs. (**c**) SPICE simulation of a cascaded 7-stage Bessel–Thompson delay filter showing Bode plot and frequency dependent delay characteristics with Fd=3.69 MHz and Fa=13.03 MHz.

Though many electronic delay methods exist, chaotic signals generally require a broad bandwidth, thus limiting selection. Physical delay lines are appropriate at higher frequencies, however, the ≈1 MHz signals require physical delay lengths that are not practical. High-fidelity digital delays are achievable but require significant power and hardware as well as sampling. In an attempt to keep the receiver design simple, low-power, and low-cost, an analog Bessel–Thompson filter circuit (shown in Figure 8a) was used. Similar all-pass filter designs have been successfully implemented with solvable chaos [46,57], however, the Bessel–Thompson filter offers a maximally flat group delay making it ideal for the delay of wideband chaotic signals [61]. As an example, the characteristics of 7 cascaded stages of the delay are demonstrated in Figure 8b with $R_{1}=6$ kΩ, $C_{1}=C_{2}=5$ pF, $R_{2}=8.2$ kΩ, $R_{3}=R_{5}=1$ kΩ, $R_{4}=3$ kΩ, and $R_{6}=3.5$ kΩ. Each stage provides 145.7 ns delay, and thus the network delays a 1.11 MHz chaotic input signal a total of 1.02 μs. A SPICE simulation of the delay’s Bode plot with frequency dependent delay characteristics are given in Figure 8c. The network has a half-power point at 19.47 MHz. We note that a slight component mismatch from the ideal Bessel–Thompson filter polynomial caused the Bode-plot response to differ slightly from the case with maximally flat group delay, however, this caused no practical issues with our hardware measurements.

### 3.3. Wireless Transmission

Next, hardware experiments verified the detection of the chaotic transmission using the design in Figure 1. First, a 6-inch baseband, wired link was provided between the fabricated chaotic oscillator and matched filter circuit. Typical waveforms and the resulting matched filter response from the baseband experiment are given by Figure 5. Although the bandwidth of the delay network was ≈20 MHz, some of the higher frequency features of $V_{u}\left(t\right)$ were lost due to filtering caused by this bandwidth restriction. Overall, it was not found to have a dramatic effect on the matched filter output as most of the signal’s power is contained below the fundamental frequency of the basis pulse, as illustrated in Figure 4.

Finally, an experiment to validate an S-band 2.45 GHz (ISM band) wireless link was performed. An illustration of the experimental procedure is given in Figure 1. The baseband signal was up/down-converted using COTS MiniCircuits RF modules. An amplitude modulation (AM) scheme was demonstrated; however, frequency modulation (FM) schemes also provide favorable results (not shown).

**Figure 9:**
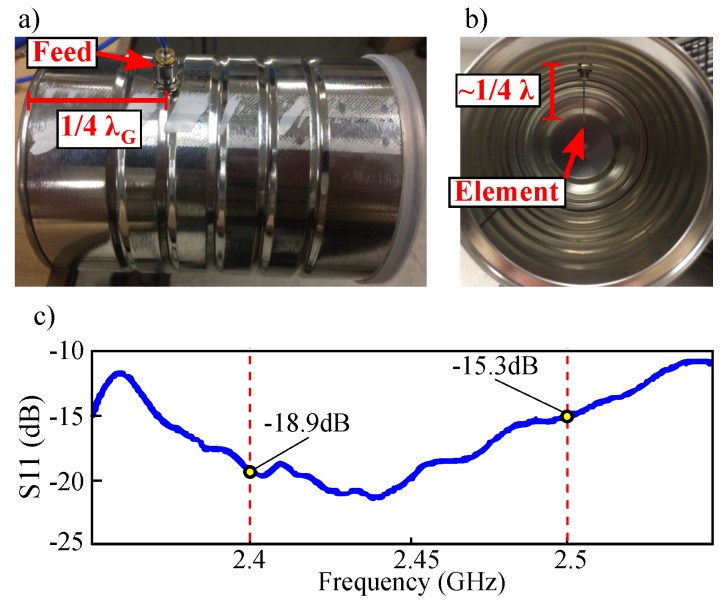
(**a**) Profile of cylindrical waveguide antenna according to the MIT coffee-can-radar instruction showing SMA feed placement $\frac{1}{4}\lambda_{G}$, where $\lambda_{G}$ is the resonant wavelength of a monopole element inside the waveguide. (**b**) Cylindrical waveguide antenna according to the MIT coffee-can-radar instruction showing the resonant monopole. (**c**) S11 plot showing typical antenna bandwidth that allowed for the transmission of the up converted chaotic signal.

The baseband chaotic signal was presented to an active 50 Ω matching network that controlled offset and amplitude of the modulating signal. The modified baseband signal was multiplied (ZX05-C60-S+) with a 2.45 GHz local oscillator (ZX-95-2536C-S+) and then amplified (ZX60-272LN-S+). The amplified signal was propagated and received using two cylindrical waveguide antennas fabricated from coffee cans as instructed in [62]. The resulting return loss over the 2.4 GHz–2.5 GHz ISM band is shown in Figure 9.

A similar receive antenna was placed 1 m from the transmitted source. The received signal was amplified (ZX60-272LN-S+) then down converted with a 2.45 GHz local oscillator (ZX-95-2536C-S+). Detection of the chaotic transmission was performed by a hardware implementation of the matched filter circuit given by Figure 5b.

## 4. Results

**Figure 10:**
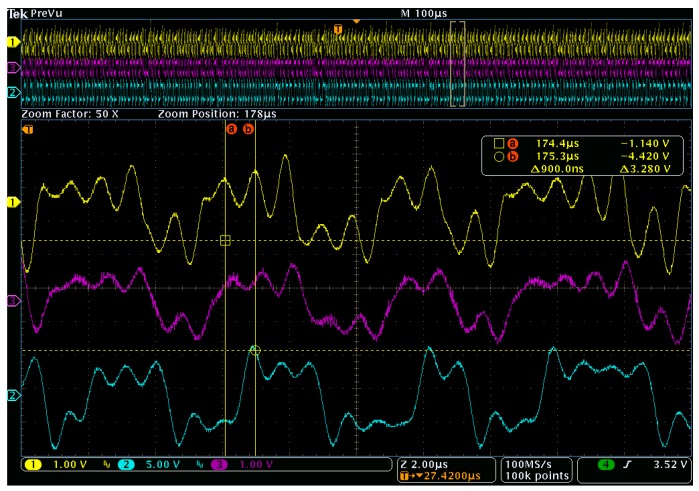
Measured data verifying the detection of a chaotic transmission showing (1) transmitted chaotic signal (top yellow), (3) attenuated and noise corrupted received signal (middle purple), and (2) matched filter output (bottom blue).

Measurements from the experiment shown in Figure 1 are given in Figure 10. The transmitted baseband signal was produced by a hardware implementation of the solvable, chaotic circuit given by Figure 6. Typical waveforms from this circuit are shown by Figure 10(1). The measured matched filter response to the chaotic oscillator transmission are provided by Figure 10(2). Measurements were made with a 1 GHz 5GS/s Tektronix MDO4104 mixed domain oscilloscope.

To quantify the results, the fundamental frequency of the basis pulse for this waveform was found by measuring the time difference between two peaks in the chaotic oscillator output. This time difference was approximately 900ns and corresponds to a fundamental frequency of 1.11 MHz. The corresponding oscilloscope measurement is provided by the top waveform (yellow) in Figure 10 with time measurements at cursor point “a” and cursor point “b”.

Measurements for the transmitted baseband, received (post down-conversion) and matched filtered signals are presented in Figure 10. The results are in close agreement with the simulated waveforms given in Figure 5.

## 5. Discussion

We have shown that a simple, RLC matched filter may be used to detect the basis function of a solvable chaotic oscillator with a fundamental frequency of ≈1 MHz. Using this concept at lower frequencies, it has previously been demonstrated that the correlation of the transmitted waveform can be produced by weighting the received and matched filtered signal using the transmitted bits $S_{n}$. Specifically, this scheme has been illustrated acoustically at 16kHz, where correlation operations used a sequence of 11 bits between a microphone array and a speaker with varying spacing of approximately 50–100 cm [19]. This arrangement allowed a consistent measurement of the time-of-flight between the transmitter and receiver. Note that [19] showed successfully matched filter reception of the transmitted chaotic signal with the noise floor raised 10dB above the transmission.

In this section, we detail an important application related to our hardware results: ranging systems. Ranging systems based on solvable chaos and matched filtering provide four main advantages: (1) naturally user-specific waveforms, (2) simple matched filter detection, (3) closed form solutions to many engineering parameters, and (4) a compressive representation that permits sub-Nyquist sampling. Further, we show that these systems can be used to address multi-user (and similarly multitarget) issues.

### 5.1. Environmental Sensing Scheme

**Figure 11:**
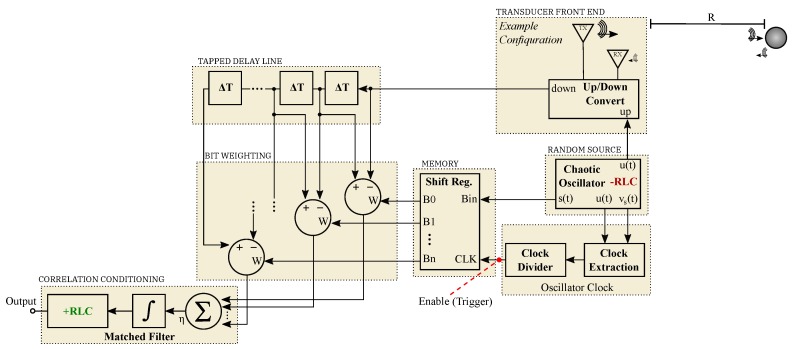
Function block diagram illustrating an environmental sensing scheme suitable for sonar and radar with applications such collision avoidance and proximity measurement. Note that a red, dotted indication of a trigger point is provided to allow for the virtual extension of correlated bits by integration gain.

A function block diagram similar to the aforementioned acoustic ranging scheme is illustrated by Figure 11. This diagram includes a generalized number of bits representing a reference waveform, a tapped delay line, and an example of an up/down conversion stage for transducer compatibility.

Operation of this environmental sensing scheme is similar to standard radar and sonar techniques and begins with the generation of a chaotic signal. A portion of this chaotic signal is propagated into the environment through the means of a transducer. A copy of the transmitted signal content is kept locally and used as a template to correlate with reflections received from the environment.

The first advantage for this scheme is found when considering the storage and sampling requirements needed to represent the transmitted waveform. Conventionally, Nyquist sampling requirements would dictate that the sample rate used to store the transmitted information must be twice the highest frequency present in the sampled signal. As mentioned earlier, this need can be costly in terms of bandwidth and power consumption.

In the case of solvable chaos, the fixed basis function serves as the continuous portion of the template used to make a correlation measurement. Practically, received signals are conditioned with delays and an integrator before being presented to a simple RLC matched filter. These delays are weighted by a symbolized version of the shift-map iterates $x_{n}$ given by the system’s guard condition events. Thus, the only data needed to be stored from the chaotic waveform are 1-bit representations of the symbols that are produced naturally by the hybrid oscillator’s discrete state. These symbols may be stored using only one bit of memory per symbol at a rate of the fundamental frequency of the oscillator. Practically, this can be realized with a simple n-bit shift register that is clocked by the guard condition events of the chaotic oscillator. The result is sub-Nyquist sampling for the generated chaotic waveforms.

**Figure 12:**
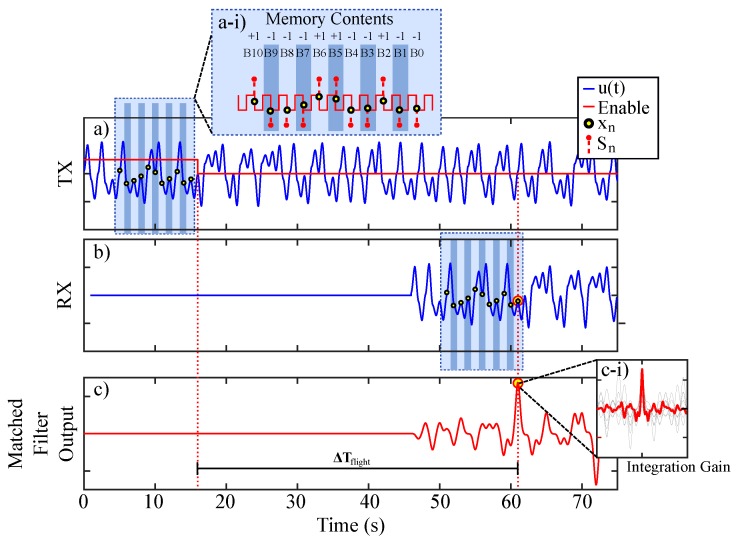
SIMULINK simulation showing (**a**) (blue) a transmitted chaotic signal, (red) an enable signal to trigger a ranging event as wells as set the system’s memory contents, (**a-i**) the system’s memory contents, (**b**) a received chaotic signal, (**c**) matched filter output, and (**c-i**) matched filter output with integration gain.

Typical signals of interest for the diagram presented in Figure 11 were produced via SIMULINK simulation and are given in Figure 12. A memory enable signal is shown in red by Figure 12a and serves as a reference for viewing an average of multiple received waveforms. This figure shows a single instance of a chaotic transmission $V_{u}\left(t\right)$.

When the enable signal is *high*, new symbols are allowed into the system’s shift register memory. Symbolic content that represents a portion of the chaotic transmission is clocked into the ranging system’s memory. An instance of the resulting symbols $S_{n}$ stored in the system’s memory contents are illustrated by Figure 12a-i. After a regular time interval, the enable signal is *low* and the stored symbols are used to weight a tapped delay line at stages that correspond to the respective bit locations in the saved bit sequence. Thus, as the transmitted signal u(t) is received, the output of the matched filter (given by Figure 12c) is processed via the stored weights and then monitored for a correlation events. Note, the matched filter is a linear filter and benefits from integration gain. Thus, the output will peak when many receptions are overlain as illustrated by Figure 12c-i. This scheme benefits from averaging, and each instance of averaging increases the effective number of correlated symbols. Thus, the averaging process provides integration gain by effectively extending the system’s memory and tapped delay line in a compact correlation receiver.

### 5.2. Multi-User Concept

Last, we connect the concepts of solvable chaos, matched filtering, and correlation receivers in an example where several of these ranging systems are operated simultaneously in a crowded environment. Spread spectrum techniques like ultra-wideband (UWB) radar systems have shown favorable results in multi-user environments [63]. Specifically, noise radars have shown multi-user compatibility for high resolution ranging measurements not only in the presence of other noise radar systems with overlapping spectra [64], but also with communication systems that share up to 30% of spectral resources before degrading radar measurements [65]. Additionally, these analyses regarding mutual interference between multiple noise radars hold when multiple chaotic radars share the same environment, where multiple systems only give a negative influence to the noise floor in a similar fashion to code division multiple access (CDMA) systems [66].

Compatibility of solvable-chaos-based ranging systems for multiple users has been demonstrated in hardware at ≈10 kHz [19] via an acoustic ranging demonstration that successfully ranged a direct path link while an identically fabricated chaotic oscillator transmitter corrupted the channel. Here, we show that more than one interferer behaves in accordance to the findings of [19] due to the random nature of these systems [67] even in the worst-case scenario of all users being phase-locked.

**Figure 13:**
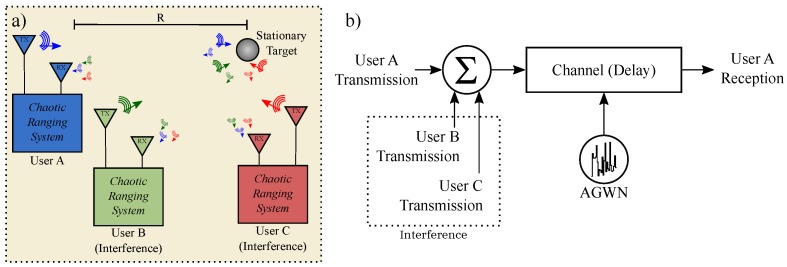
(**a**) Illustration of a crowded sensing enviornment where user “*A*” suffers from interference due to users “*B*” and “*C*”. (**b**) Channel model for SIMULINK experiment showing the detection of transmissions from user “*A*” that includes interference from users “*B*” and “*C*”, AGWN and a delay corresponding to a ranging measurement.

Consider the crowded scenario illustrated by Figure 13a, where a particular user “*A*” of interest is making range measurements of a stationary target at a distance of *R*. Consider the following. User “*A*” transmits a waveform to measure distance, however, user “*A*” also receives transmissions generated by other users (“*B*” and “*C*”). As discussed earlier, FMCW schemes require new slopes to form a chirp sequence [29] with randomization in order to minimize mutual interference [15]. Here, we show that solvable chaos-based ranging efficiently generates unique signals that may be optimally detected and mitigate against interference/jamming.

A SIMULINK simulation of the detection of solvable, chaotic transmissions from user ‘*A*’ that includes interference from users ‘*B*’ and ‘*C*’, AGWN and a delay corresponding to a ranging measurement was performed as illustrated in Figure 13b.

**Figure 14:**
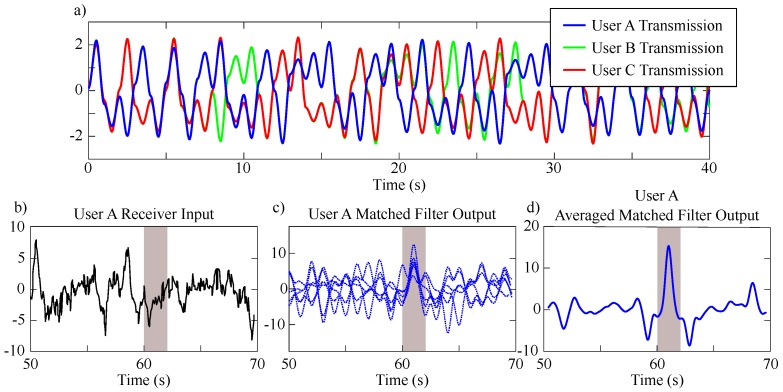
(**a**) Time series SIMULINK simulation data of solvable chaotic transmissions from three identical users: ‘*A*’ (blue) with initial condition of u(0)=0.2, ‘*B*’ (green) with initial condition of u(0)=0.201 and ‘*C*’ (red) with initial condition of u(0)=0.202 showing the divergence of their transmissions due to small changes in their initial conditions. (**b**) Received signal by user ‘*A*’, prior to its matched filter, that has been corrupted by noise and as well as interference from the signals of users ‘*B*’ and ‘*C*’. (**c**) Example of six different matched filter outputs from user ‘*A*’. (**d**) Output from the correlation receiver of user ‘*A*’ via the averaging and integration gain from the six signals from (**c**).

When considering three identical users all ranging with solvable chaos, each user will have a unique transmitted waveform despite identical hardware. This comes from each chaotic system being sensitive to initial conditions; even if each user was given the same initial conditions or start-up conditions for their circuits, each transmission would diverge from one another due to small differences. This divergence is shown if Figure 14a where the output from users: “*A*” (with initial condition of u(0)=0.2), “*B*” (with initial condition of u(0)=0.201) and “*C*” (with initial condition of u(0)=0.202) are shown to overlap only for short time until they begin to diverge. On a long enough timescale the information content in these transmissions is statistically orthogonal despite starting at similar initial conditions [39]. Furthermore, even in the unpractical case for identical hardware and initial conditions, noise is pervasive in all physical systems and will cause subsequent states, no matter how close, to diverge exponentially in each oscillator. Practically, this means that multi-user transmissions will diverge due to minute parameter variations no matter the starting conditions.

To showcase solvable chaos, we select the worst case for interference, which arises when all three users are phase-locked as shown in Figure 14a. Though this is unlikely to occur in practice unless strong coupling is provided, we chose this worst case scenario to demonstrate that the tapped-delay-line matched filter scheme shown in Figure 11 is robust to this type of mutual interference.

A channel containing a transmission from user “*A*” was corrupted by noise and two chaotic interferers, as shown in Figure 14b. The voltage magnitude of the simulated user “*A*” transmission and each interferer was 2.32 V. The voltage magnitude of SIMULINK’s AGWN function was 4V with a power spectral density of 0.1 (W/Hz) and a correlation time of 0.1 s. The resulting SNR in the simulated channel was −11.42 dB.

The resulting, noisy reception shown by Figure 14b was detected using user ‘*A*’s matched filter with 10 symbols weighted in a tapped delay line. An overlay of resulting matched filter responses is given by Figure 14c. Each response shares a common peak while the values on either side tend to vary. A total of six returns were measured starting with an initial condition u(0)=0.1 V and ending with u(0)=0.6 V in steps of 0.1 V. The interferers were kept to have the same respective initial conditions of 0.201 V and 0.202 V for each iteration. As the matched filter output from the 6 instances of the 10-symbol noise ranging system are averaged, the system is virtually extended to a 60-symbol noise ranging system. The resulting correlation peak due to this integration gain is shown by Figure 14d.

## 6. Conclusions and Future Work

**Figure 15:**
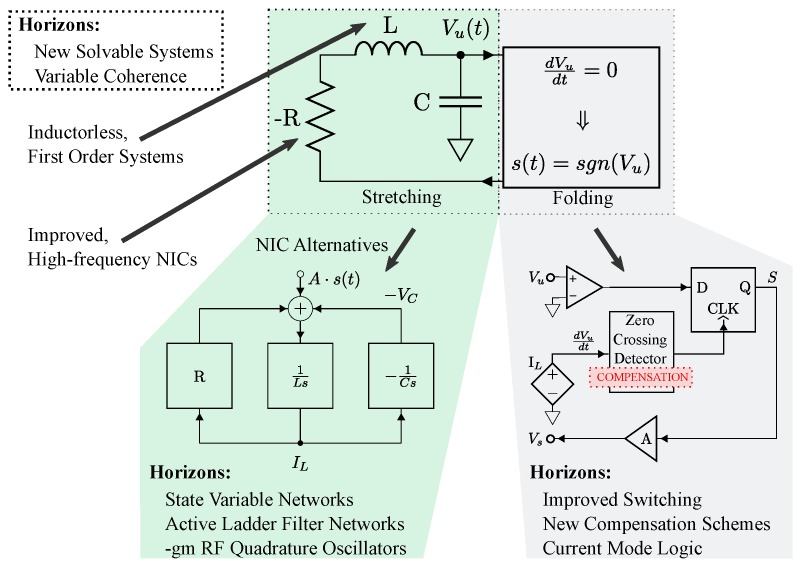
Exploded function block diagram showing potential integrated circuit design techniques for solvable, chaotic oscillators for stretching & folding portions of the chaotic oscillator circuit to obtain higher bandwidth for the baseband chaotic transmission signal.

At this point, we hope the reader has appreciated the potential benefits of solvable chaos for low-cost ranging systems. Moving forward, applications may require increased bandwidth to achieve high range resolution [68]. For range resolutions on the order of 10 to 100 cm, bandwidths on the order of 100 MHz to 1 GHz are desirable for many ranging schemes. One way to achieve these range resolutions is to increase the operational bandwidth of the baseband chaotic transmission through the advantage of the closed-form solution which can yield compensation techniques [17] and through microelectronic integration [46,60,69]. Other techniques show promise in overcoming the frequency limitations of negative impedance converters by considering synthesized ladder filter designs [46,60] and negative impedance converter compensation schemes [59]. These considerations and speculative lines of inquiry are illustrated by Figure 15.

Horizons to expand applications of solvable ranging systems via signal processing include direct velocity measurement techniques via Doppler effects. For example, this capability would promote solvable-chaos-based automotive radars beyond simple obstacle detection to applications involving higher speeds. Simple approaches to enabling Doppler capability in solvable systems may involve a swept basis function or a bank of simple +RLC matched filters.

Further, many areas outside of autonomous mobile robots and transportation are growing in applications related to IoT. Healthcare, for example, is expanding in regard to wireless sensors and communication. As this application space matures a clear need for spectral management will arise to support many sensors [70]. Spectral management for wireless sensors would enable multiple patients to be monitored for critical activity via remote sensing [71]. Feasibility of such sensors has been demonstrated through patient fall detection [3], health monitoring using through-the-wall UWB radars for heart and breathing rates [72], and even the identification of abnormalities in the detected heart and breathing rates such as heart rate variability (HRV) and respiratory sinus arrhythmia (RSA) [73]. The nature of UWB and noise radars paired with these types of measurements additionally enables many sensors in the same local environment to cooperate in the search for survivors in disaster relief scenarios [74]. Considering these motivations, the RF validation of many solvable systems utilized for wireless sensing is an important and exciting direction.

In summary, we have demonstrated a simple, inexpensive RLC matched filter for a chaotic noise source at radio frequencies. Key components for solvable, chaos-based ranging at an operational frequency of 1.11 MHz were first verified over a wired channel and then demonstrated using an S-band 2.45 GHz wireless channel. Measured data agreed with theory and simulation. Additionally, we have shown the general multi-user feasibility of several solvable chaotic ranging systems even for the worst case scenario of phase-locked, mutual interferers. These results represent progress towards low cost, high speed, and spread spectrum ranging systems for multi-user, crowded environments.

## Abbreviations

The following abbreviations are used in this manuscript:

AGWN	Additive Gaussian white noise
AM	Amplitude modulation
BER	Bit error rate
BPSK	Binary phase-shift keying
CDMA	Code division multiple access
COTS	Consumer off-the-shelf
CW	Continuous wave
DSP	Digital signal processor
DOAJ	Directory of open access journals
FFT	Fast Fourier Transform
FM	Frequency modulation
FMCW	Frequency modulated continuous wave
FPGA	Field programmable gate array
IoT	Internet of Things
MDPI	Multidisciplinary Digital Publishing Institute
NIC	Negative impedance converter
op-amp	Operational Amplifier
RF	Radio frequency
RLC	Resistor, inductor, capacitor
-RLC	Negative resistor, inductor, capacitor
SNR	Signal-to-noise Ratio
UWB	Ultra-wideband
